# Bortezomib Prescription Pattern for the Treatment of Multiple Myeloma by Hematologists in Nigeria

**DOI:** 10.1200/JGO.17.00033

**Published:** 2017-11-01

**Authors:** Kaladada I. Korubo, Anazoeze J. Madu, Helen C. Okoye, Benedict Nwogoh

**Affiliations:** **Kaladada I. Korubo**, University of Port Harcourt, Rivers; **Anazoeze J. Madu** and **Helen C. Okoye**, University of Nigeria Nsukka, Enugu; and **Benedict Nwogoh**, University of Benin, Benin City, Edo, Nigeria.

## Abstract

**Purpose:**

Novel therapy has dramatically changed the outcome of patients with myeloma. Current National Comprehensive Cancer Network guidelines give bortezomib-based combinations a central role in the management of multiple myeloma (MM). The aim of this survey is to assess the use of bortezomib for the treatment of MM by hematologists practicing in Nigeria.

**Materials and Methods:**

This is a cross-sectional observational survey. A structured, prevalidated questionnaire was self-administered to different cadres of hematologists. Data collected were analyzed using SPSS software version 21 (IBM, Chicago, IL).

**Results:**

There were 54 respondents from 24 centers across the country. The most frequently used drugs for first-line therapy were thalidomide (66.7%), dexamethasone (54.2%), and bortezomib (48%), and a combination of bortezomib, thalidomide, and dexamethasone (16.7%) was the most frequently used first-line regimen. Of the 54 hematologists, 39 (72.2%) had prescribed bortezomib previously; no one had used bortezomib as monotherapy. Drug unavailability (86.7%) and cost (46.7%) were the major reasons for those who had not prescribed bortezomib. Approximately 56.4% of responders had patients who had experienced adverse effects, of which neuropathy was the most common (86.3%).

**Conclusion:**

Bortezomib, thalidomide, and dexamethasone was the most frequently used first-line regimen to treat myelomatosis. Thalidomide and dexamethasone were the most frequently used drugs in myeloma treatment. Despite poor access to health care, coupled with the high cost and poor availability of bortezomib in our low- or middle-income country, those who prescribed bortezomib did so frequently (in more than half of their patients).

## INTRODUCTION

Multiple myeloma (MM) is a B-cell malignancy of plasma cells resulting in bone marrow or tissue proliferation of these cells associated with anemia, renal impairment, bone disease, and hypercalcaemia.^1,2^ The disease is twice as common in people of African descent compared with whites and is the most common hematologic malignancy in blacks, with males being more affected than females.^3^ Although the median age for developing MM is approximately 65 years^4^ in the Western world, in Nigeria the median age is lower (54 to 60 years).^5-7^ It makes up approximately 10% of hematologic malignancies and 1% of total cancers.^2,3^

Since the late 1960s, the standard therapy for MM was melphalan and prednisolone. By the early 1980s, hematopoietic stem cell transplantation (HSCT) was used in younger patients with MM. For a long while there was stagnation in development of new therapies for MM, especially for patients not eligible for transplantation; however, a new era began in the late 1990s, when novel drugs such as thalidomide (an immune modulator) and bortezomib (the first proteasome inhibitor) were approved for the treatment of MM. These and other novel agents have brought about a dramatic improvement in both the response rate and overall survival in patients with myeloma.^8^

Bortezomib is a dipeptide boronic acid analog that was first approved for the treatment of MM in 2003. The mechanism of action involves reversible inhibition of proteasome activity in the 26s proteasome complex, leading to accumulation of misfolded proteins in the endoplasmic reticulum of the myeloma cells, thereby causing DNA-induced cell death. The drug also inhibits the mitogen-activated kinase and nuclear factor–κB pathways, which are both important in survival of the myeloma cell. Bortezomib acts rapidly to reduce the *m*-protein and free light chains concentration.^9-11^ It is not excreted by the kidneys and is advantageous in patients with MM who may present with renal impairment, as is the case in up to 40% of patients with myeloma.^12,13^ The drug is a front-line therapy for myeloma and is indicated in patients with myeloma either eligible or ineligible for transplantation.

Therapeutic options for MM are limited in Nigeria because novel drugs are not readily available and are high priced. The majority of patients make out-of-pocket payments for chemotherapy prescriptions, and noncompliance due to financial factors is rife. Moreover, facilities for HSCT to treat hematologic malignancies are not yet available. Melphalan-based therapy largely remains the standard regimen used, because it is affordable and available; it has been used even in younger patients who are ineligible for transplantation.^5,14,15^ This implies suboptimal management and consequent reduction in treatment outcomes for this group of patients. In scenarios where bortezomib is used, the dosing pattern may be irregular or adulterated to make for affordability, thereby resulting in underdosing of patients. Presently, there are no national guidelines for the management of MM in Nigeria. The aim of this survey is to assess the use of bortezomib for the treatment of MM by hematologists practicing in Nigeria.

## MATERIALS AND METHODS

**Fig A1:**
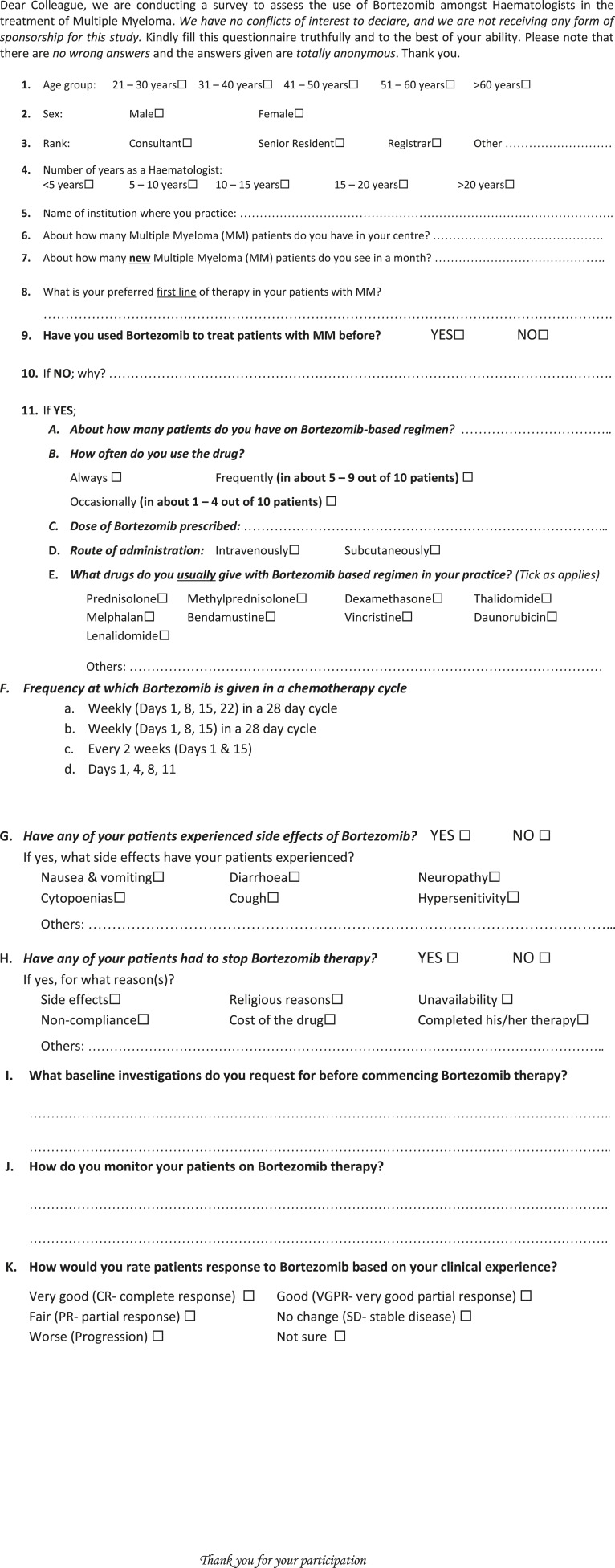
Bortezomib prescription pattern for the treatment of multiple myeloma by hematologists in Nigeria.

This is a cross-sectional observational survey conducted during the 2016 annual scientific conference of the Nigerian Society of Haematology & Blood Transfusion. A structured, prevalidated questionnaire (Appendix Fig A1) was self-administered to different cadres of hematologists who attended the meeting. Data collected were analyzed using SPSS software version 21 (IBM, Chicago, IL). Results were expressed in means and percentages and illustrated as figures and tables.

## RESULTS

**Fig 1:**
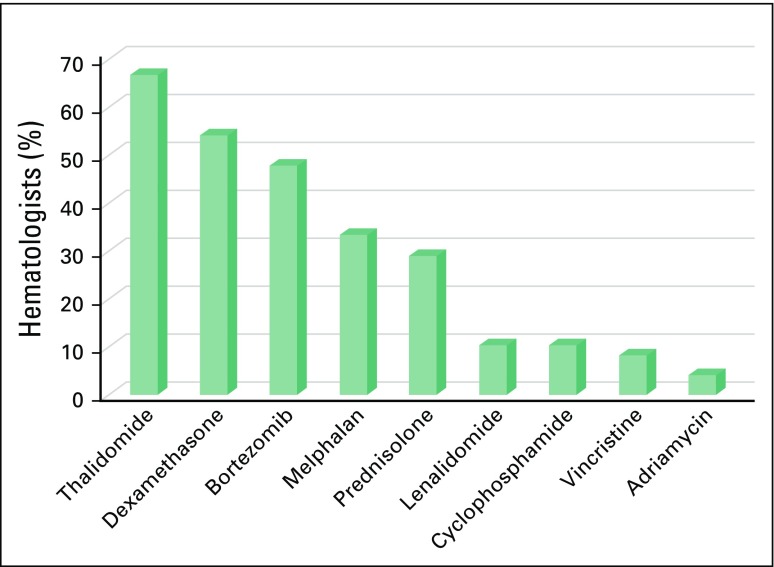
Drugs used as part of first-line therapy in multiple myeloma.

**Fig 2:**
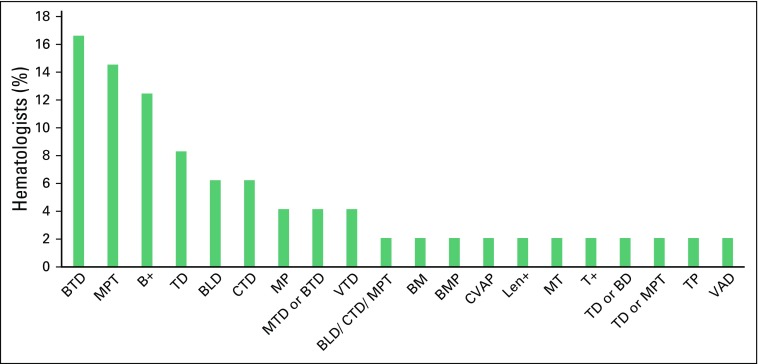
First-line chemotherapy regimen used by hematologists for the treatment of multiple myeloma. B+, bortezomib based; BD, bortezomib, dexamethasone; BLD, bortezomib, lenalidomide, dexamethasone; BM, bortezomib, melphalan; BMP, bortezomib, melphalan, prednisolone; BTD, bortezomib, thalidomide, dexamethasone; CTD, cyclophosphamide, thalidomide, dexamethasone; CVAP, cyclophosphamide, vincristine, doxorubicin, prednisolone; Len+, lenalidomide based; MP, melphalan, prednisolone; MPT, melphalan, prednisolone, thalidomide; MT, melphalan, thalidomide; MTD, melphalan, thalidomide, dexamethasone; T+, thalidomide based; TD, thalidomide, dexamethasone; TP, thalidomide, prednisolone; VAD, vincristine, doxorubicin, dexamethasone; VTD, vincristine, thalidomide, dexamethasone.

There were 54 respondents from 24 centers across the country. Their ages ranged from 21 to 60 years; 29 (53.7%) were women and 25 (46.2%) were men. The estimated average number of myeloma cases per center was 12.1 ± 11.4 (median, 10 cases per center), with an average of 2 ± 1.8 new cases seen per month. Regarding first-line therapy, thalidomide was the most frequently used drug (66.7%), followed by dexamethasone (54.2%; Fig 1), and the most frequently used first-line regimen was a combination of bortezomib, thalidomide, and dexamethasone (BTD; Fig 2). Of the 54 hematologists, 39 (72.2%) had prescribed bortezomib previously, whereas 15 (27.8%) had never used bortezomib for the treatment of MM. Reasons for not prescribing the drug included unavailability of the drug in their locality (n = 13; 86.7%) and high cost of the drug (n = 7; 46.7%). One respondent (6.7%) had not yet prescribed the drug because he/she was yet to have a patient with MM, and one respondent did not give specific reason(s) for not prescribing bortezomib.

For the 39 respondents who had used bortezomib previously in the treatment of myeloma, the average number of patients who were currently receiving bortezomib therapy was 5.5 (± 6). The frequency of use of bortezomib by the hematologists differed: two (5.1%) always used bortezomib, the majority (n = 20; 51.3%) frequently prescribed the drug (in approximately five to nine out of 10 patients), 13 (33.3%) occasionally used it (in approximately one to four out of 10 patients), and four (10.2%) did not specify the frequency at which they prescribed the drug. None of the hematologists used bortezomib as monotherapy. The drug most frequently used with bortezomib was dexamethasone (74.4%), followed by thalidomide (53.9%). Others were prednisolone (28.2%), lenalidomide (18%), melphalan (10.3%), vincristine (2.6%), and doxorubicin (2.6%). The dose of bortezomib prescribed varied among the responders, as did the duration of the cycle. Ten (25.7%) prescribed the dose of 1.3 mg/m^2^ for their patients, and another 10 (25.7%) prescribed 2 mg. One responder administered a dose of 1 mg to patients, but seven (17.9%) could not remember the dose of bortezomib prescribed and 11 (28.2%) did not respond. Bortezomib was administered on days 1, 4, 8, and 11 in a 21-day cycle by 13 (35.9%) hematologists; weekly (days 1, 8, 15, and 22) in a 28-day cycle by nine (23.1%); weekly (days 1, 8, and 15) in a 28-day cycle by eight (20.5%); and every 2 weeks (days 1 and 15) by three (7.7%). The drug was administered through the intravenous route by the majority (n = 25; 64.1%) of the responders; nine (23.1%) administered it via the subcutaneous route, and two (5.1%) used either the intravenous or subcutaneous routes.

More than half (56.4%) of those who prescribed bortezomib had patients who had experienced adverse effects of the drug, which included neuropathy (n = 19; 86.3%), nausea (n = 8; 36.3%), vomiting (n = 7; 31.8%), cytopenias (n = 6; 27.3%), diarrhea (n = 1; 4.5%), and cough (n = 1; 4.5%). There were 16 (41%) participants whose patients had discontinued bortezomib therapy, and the major reason was cost of the drug (n = 12, 75%). Other reasons for stopping bortezomib in their patients included adverse effects (n = 5; 31.2%), completion of therapy (n = 4; 25%), and noncompliance by the patient (n = 3; 18.8%). The full blood count was the most common investigation requested, both at baseline and during monitoring of therapy. Other investigations requested by the hematologists are listed in Table 1. When asked their assessment of patients’ response to bortezomib, 12 (30.8%) did not comment. Of the 27 who commented, 15 (55.6%) believed that their patients had a complete response to therapy, five (18.5%) believed they had very good partial response, and another five (18.5%) believed they had partial response to bortezomib therapy. Two responders (7.4%) were not sure of the response of their patients to bortezomib.

**Table 1:**
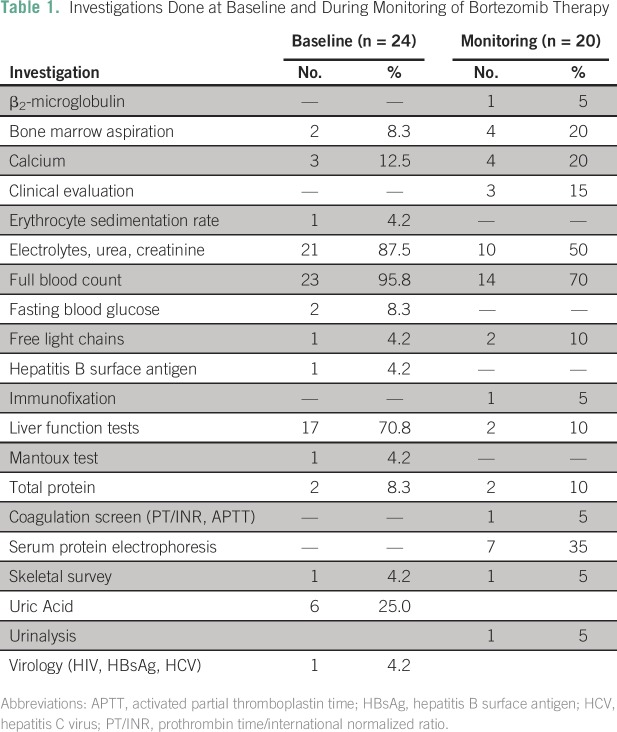
Investigations Done at Baseline and During Monitoring of Bortezomib Therapy

## DISCUSSION

The annual scientific meeting of the Nigerian Society of Haematology & Blood Transfusion offers an opportunity for hematologists from different parts of the country to come together. Our survey showed that all hematologists practiced a combination therapy for MM treatment. A little more than one fourth of hematologists had never prescribed bortezomib, and this was mostly due to unavailability of the drug in their locality and cost of the drug. Current National Comprehensive Cancer Network guidelines for treatment of myeloma give bortezomib-based combinations a central role in the management of MM. This implies that approximately one fourth of the patients with myeloma received suboptimal drug therapy for varied reasons.

Of the novel agents in myeloma therapy, thalidomide, bortezomib, and lenalidomide were the only drugs that had been used by some of the hematologists. This is in consonance with best practice, but the number of patients receiving these compounds is quite low. More work needs to be done in the area of accessibility and subsidization of these newer molecules to affect the management of patients with myeloma in our environment. The use of health insurance schemes is poorly developed and still inadequate in Nigeria, with > 90% of the population uninsured.^16,17^ For the few insured patients, oncology services are not yet covered. Therefore, payment for medical services in Nigeria is usually out of pocket, and for a country where the majority of the population lives on < $1 per day, the unavailability and cost of these novel agents, which are highly effective in the treatment of MM, add to the burden of this disease in our environment. Bortezomib was first approved for the treatment of MM in 2003 and is the first in class of the proteasome inhibitors. However, almost 15 years later, the drug is still not readily available, nor is it affordable for most patients, especially because they have to pay out of pocket for it.

Despite the challenges with access to bortezomib, it is encouraging that the old standard of care using melphalan and prednisolone is on the decline; this regimen was the seventh in the line of first-line regimens used by the hematologists. Thalidomide was the most widely used novel agent, followed by bortezomib, with only a few prescribing lenalidomide. This is understandable, because thalidomide is a component of several regimens including melphalan or bortezomib. The frequency with which these novel agents are prescribed may be directly attributed to the cost, with lenalidomide being the most expensive and thalidomide the most affordable of the three. None of the hematologists had prescribed other newer novel agents, such as carfilzomib, pomalidomide, daratumumab, or ixazomib, indicating that these drugs are still unavailable in our environment.

Novel agents lead to remarkably better overall survival and outcome in MM, with patients living longer.^18^ The dramatic response to bortezomib and other novel agents has led researchers to question if these drugs should replace or delay the need for autologous HSCT in younger patients with myeloma who are ineligible for transplantation.^19^ Novel drugs will be of great benefit, because HSCT for the treatment of hematologic malignancies is not yet being practiced in our country.

The dose of bortezomib is 1.3 mg/m^2^ administered intravenously or subcutaneously either on days 1, 4, 8, and 11 or weekly; however, the hematologists in our study could not seem to agree on the dose or the duration it is administered in a cycle. Altogether, more hematologists prescribed bortezomib weekly in a 28-day cycle (either on days 1, 8, 15, and 22 or days 1, 8, and 15) than in a 21-day cycle. Bortezomib is supplied as either 2-mg or 3.5-mg powder for intravenous or subcutaneous use. It may be that some of the hematologists prescribed 2 mg irrespective of their patient’s body surface area because of the cost of the drug, thereby avoiding use of the 3.5-mg preparation, which is even more expensive than the 2-mg preparation. The majority of the hematologists administered the drug via the intravenous route. This may have led to quicker onset of neuropathy, which was experienced by the majority of their patients.

One of the major adverse effects of bortezomib therapy is neuropathy, which is of different grades and sometimes severe enough to warrant discontinuing the drug. Our study shows that neuropathy was the most common adverse effect experienced by the responders’ patients, and approximately one third of the hematologists had patients who had to discontinue the drug because of unbearable adverse effects.

In conclusion, since the late 1990s, novel therapies in MM have become game changers in the treatment of MM. BTD was the combination most frequently used as first-line therapy by most hematologists to manage myelomatosis. Thalidomide and dexamethasone were the most frequently used drugs in myeloma treatment, and among the physicians who used bortezomib, most of them used it frequently. Although there is poor access to health care and high cost and poor availability of bortezomib for the management of MM in our low- or middle-income country, those who prescribed bortezomib did so frequently, and melphalan plus prednisolone is no longer the standard of care among hematologists.

There were limitations to our study. This survey did not take into consideration the number of cycles of bortezomib therapy prescribed by the hematologists. Also, severity of toxicity to bortezomib was not recorded.
